# Mid-Diastolic Events (L Events): A Critical Review

**DOI:** 10.3390/jcm10235654

**Published:** 2021-11-30

**Authors:** Emanuele Di Virgilio, Francesco Monitillo, Daniela Santoro, Silvia D’Alessandro, Marco Guglielmo, Andrea Baggiano, Laura Fusini, Riccardo Memeo, Mark G. Rabbat, Stefano Favale, Matteo Cameli, Andrea Igoren Guaricci, Gianluca Pontone

**Affiliations:** 1 University Cardiology Unit, Polyclinic University Hospital, 70124 Bari, Italy; emanueledrit@gmail.com (E.D.V.); dr.francescomonitillo@gmail.com (F.M.); danina2012@gmail.com (D.S.); dottor.riccardomemeo@gmail.com (R.M.); stefano.favale@uniba.it (S.F.); 2 University Neurology Unit, Polyclinic University Hospital, 70124 Bari, Italy; silv.dalessandro@gmail.com; 3 Centro Cardiologico Monzino, IRCCS, 20138 Milan, Italy; marco.guglielmo@cardiologicomonzino.it (M.G.); andrea.baggiano@cardiologicomonzino.it (A.B.); laura.fusini@cardiologicomonzino.it (L.F.); gianluca.pontone@cardiologicomonzino.it (G.P.); 4 Department of Medicine and Radiology, Division of Cardiology, Loyola University of Chicago, Chicago, IL 60660, USA; MRABBAT@lumc.edu; 5 Edward Hines Jr. VA Hospital, Hines, IL 60141, USA; 6 Department of Medical Biotechnologies, Division of Cardiology, University of Siena, 53100 Siena, Italy; matteo.cameli@yahoo.com

**Keywords:** echocardiography, diastolic function, diastasis, L wave, speckle tracking imaging

## Abstract

Mid-diastolic events (L events) include three phenomena appreciable on echocardiography occurring during diastasis: mid-diastolic transmitral flow velocity (L wave), mid-diastolic mitral valve motion (L motion), and mid-diastolic mitral annular velocity (L’ wave). L wave is a known marker of advanced diastolic dysfunction in different pathological clinical settings such as left ventricle and atrial remodeling, overloaded states, and cardiomyopathies. Patients with L events have poor outcomes with a higher risk of developing heart failure symptoms and arrhythmic complications, including sudden cardiac death. The exact mechanism underlying the genesis of mid-diastolic events is not fully understood, just as the significance of these events in healthy young people or their presence at the tricuspid valve level. We also report an explicative case of a patient with L events studied using speckle tracking imaging of the left atrium and ventricle at the same reference heartbeat supporting the hypothesis of a post-early diastolic relaxation or a “two-step” ventricular relaxation for L wave genesis. Our paper seeks to extend knowledge about the pathophysiological mechanisms on mid-diastolic events and summarizes the current knowledge.

## 1. Introduction

**Figure 1:**
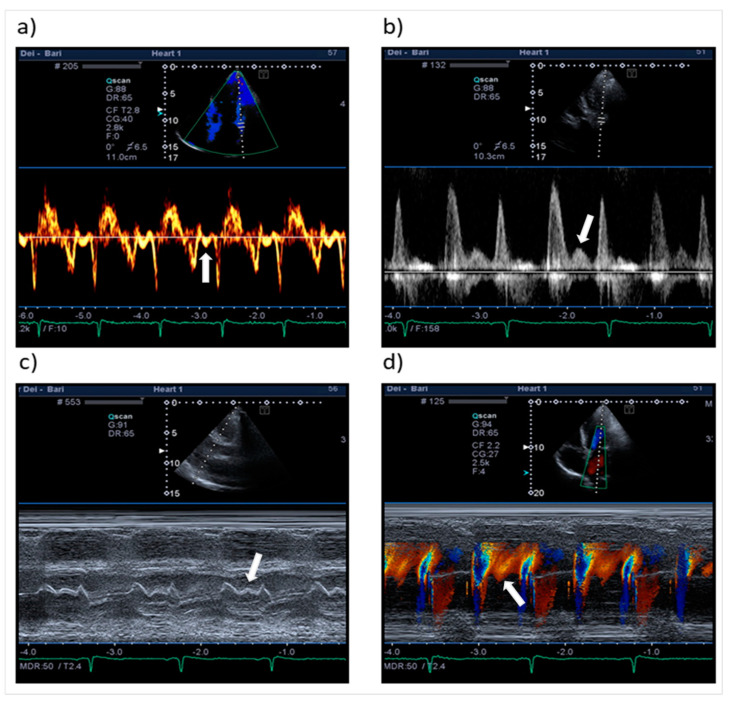
Mid-diastolic events. (**a**) Mid-diastolic mitral velocity at the level of lateral annulus (L’ wave). (**b**) Mid-diastolic transmitral flow (L wave). (**c**) Mid-diastolic mitral valve motion (L motion). Note the “W” shape of the anterior mitral leaflet motion. (**d**) Mid-diastolic transmitral flow and right upper pulmonary vein flow in color M-mode sampling. The white arrows indicate L events.

**Figure 2:**
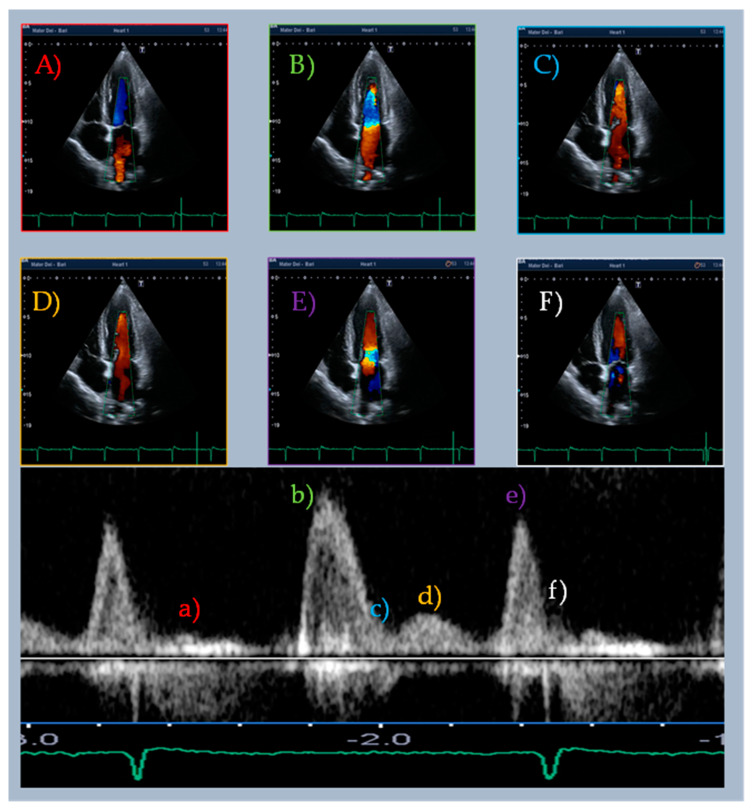
Color Doppler imaging for the analysis of the L wave and flow pathway. The upper panels (**A**–**F**) show the transmitral and right upper pulmonary vein flow sampled simultaneously in the color Doppler apical four-chamber view. The lower panel shows the corresponding transmitral flow sampled in pulsed-wave Doppler (a–f). (**A**) (a) Systolic phase: anterograde pulmonary vein flow and mitral valve closure. (**B**,**C**) Early diastolic phase: anterograde mitral and pulmonary vein flows, (b,c) E wave. (**D**) Diastasis: transmitral mid-diastolic flow and continuous pulmonary vein flow, (d) L wave. (**E**) End-diastolic phase: increased mitral flow velocity and no pulmonary vein flow, (e) A wave. (**F**) Early systolic phase: mitral valve closure.

Recently, echocardiography and other diagnostic techniques in the field of cardiovascular diseases have been equipped with new application tools [1,2,3,4,5,6,7,8]. This constant evolution should not overshadow old diagnostic techniques that embrace crucial pathophysiological principles. The study of diastasis and mid-diastolic events initially appeared in the literature in the 1950s [9]. During the sinus rhythm (SR), diastasis (from ancient Greek διάστασις, “separation”) is the diastolic phase between early rapid filling and atrial systole; it is considered a quiescent stage of the diastole due to the small gradient and low flow between cardiac chambers. Nonetheless, occasionally, we can observe mid-diastolic events that we call “L events” during the echocardiographic examination (Table 1, Figure 1). Among these, mid-diastolic transmitral flow velocity (L wave) is the most studied [10,11,12,13,14] (Figure 2). It was called “L” because it follows the K and J pulmonary vein flow phases (corresponding to the S and D waves of pulmonary venous flow Doppler sampling). Although L waves were first reported in healthy individuals, many studies indicate their presence as a marker of advanced diastolic dysfunction and increased risk of adverse cardiac events [15,16,17,18]. L events are affected by heart rate (HR), cardiac rhythm, and loading conditions. The exact pathophysiology of this flow is still not clear. This article aimed to critically review the existing literature on mid-diastolic events (especially L waves) in different clinical scenarios.

## 2. Epidemiology

The studied populations were heterogeneous, mostly composed of aged patients having different underlying cardiovascular disease (CVD) with no significant valvular heart disease (VHD) (Table 2). Although most of the subjects featured several cardiovascular risk factors, there was no consistent significant association with L events.

Since well-represented diastasis is a fundamental prerequisite, L events are associated in clinical scenarios with a low HR and the prevalence seems higher in patients with atrial fibrillation (AF). Ha et al. reported a prevalence of triphasic transmitral flow of 0.9% in a sample of 9004 subjects in SR who underwent echocardiography examination in a time interval of 9 months [19]. A subsequent publication found a low rate of L events in a heterogeneous group of patients in SR, but a much higher rate among those with AF (2.4% vs. 34%, *p* < 0.001) [15]. In this report, those in AF expressing an L wave are older and more frequently female.

**Table 2:** Characteristics of patients in previous studies.

Author/Year	Study	Population Studied	Age (Year)	N	% L Wave (+)	% Female L Wave (+)
Ha, 2006 [13]	Observational	Patients with L wave (83) from all the patients in SR (n = 9004) examined in the same period	63 ± 10	9004	0.9	60
Nakai, 2007 [15]	Observational	Patients with persistent nonvalvular AF. Patients in SR in the same period (for prevalence estimation of the L wave)	L wave (+): 81 ± 8 L wave (−): 73 ± 9 All in AF	945 (SR) 99 (AF)	2.4 (SR) 34 (AF)	68
Lam, 2008 [20]	Observational	Patients in SR with LVH and preserved LVEF, without significant VHD	65 ± 11	177	20	63
Ho, 2013 [17]	Observational	Patients with persistent nonvalvular AF	70 ± 10	196	-	-
Ari, 2013 [14]	Observational	Patients with persistent nonvalvular AF	L wave (+): 65.45 ± 6.45 L wave (−): 62.04 ± 7.79	70	31	73
Kim, 2017 [12]	Observational	Patients in SR with L wave without significant VHD and normal LVEF from all (n = 20,854) the patients with diastolic dysfunction	63 ± 12	144	1	61
Masai, 2018 [16]	Observational	Patients with HF. No significant VHD, no FA or frequent EB or HR > 120 bpm	70 ± 15	151	32	?
Sugiura, 2019 [10]	Observational retrospective	Patients with HCM with low SCD risk factor and functional class NYHA I–II	58 ± 13	96	15	43
Saito, 2020 [11]	Observational retrospective	Patients with the first clinical diagnosis of HCM	L wave (+): 49 ± 18 L wave (−): 52 ± 15	445	32	44

SR: sinus rhythm; AF: atrial fibrillation; LVH: left ventricle hypertrophy; VHD: valvular heart disease; LVEF: left ventricle ejection fraction; HF: heart failure; EB: extrasystolic beats; HR: heart rate; HCM: hypertrophic cardiomyopathy; SCD: sudden cardiac death; NYHA: New York heart association.

Regardless of the basal rhythm, two groups of patients reach the highest rate of prevalence of L events: left ventricle hypertrophy (LVH), including hypertrophic cardiomyopathy (HCM), and heart failure (HF) patients. In 2008, Lam et al. reported in a population of subjects in SR with LVH and preserved left ventricular ejection fraction (LVEF) a prevalence of triphasic transmitral flow and mid-diastolic mitral annular velocity (L’ wave) patterns of 20% and 30%, respectively [20]. In a sample of younger subjects affected by HCM with a low sudden cardiac death (SCD) risk score and normal LVEF, the reported rate of L wave prevalence was 15.1% [10]. Recently, in a cohort of 445 patients (both in SR and AF) with the first clinical diagnosis of HCM, Saito et al. reported a higher prevalence of the L wave (32.4%). In the same population, L wave-positive subjects were more frequently female and with AF [11].

In partial discordance with previous studies, Kim et al. noted a triphasic transmitral flow pattern in only 1% of an entire population of 20,845 subjects with SR and any degree of diastolic dysfunction [12]. The authors did not describe the characteristics of the entire population but it seems that diastolic dysfunction per se was not sufficient for the appearance of L events.

The relationship with HF is bidirectional since patients with an L wave (in both AF or SR) more frequently had HF (and were at risk of developing HF-related symptoms) and the HF subjects more frequently expressed a triphasic transmitral pattern. A high prevalence of the L wave was reported in both groups of patients with preserved and reduced LVEF (36% of heart failure cases with preserved ejection fraction (HFpEF) and 28% of heart failure cases with reduced ejection fraction (HFrEF)) during SR [16]. In a previous report enrolling patients affected by markedly depressed LVEF, only 10% of dilated cardiomyopathy subjects had a triphasic transmitral pattern. [21].

There are no epidemiologic data about L events in young healthy people so prevalence in this group remains unknown.

## 3. Pathophysiology

**Figure 3:**
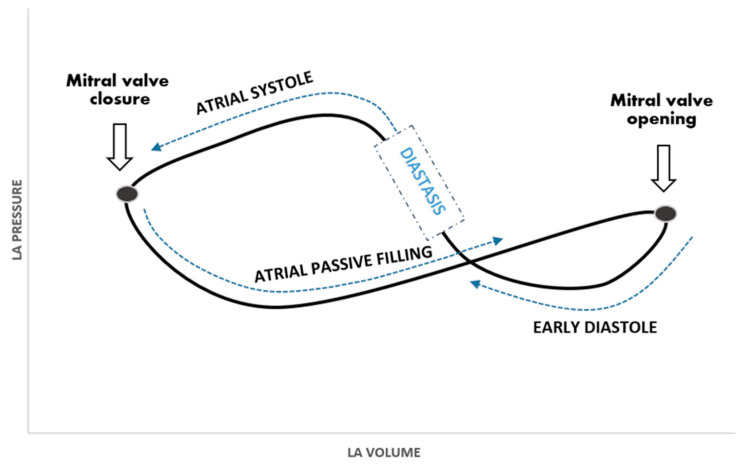
Left atrial pressure–volume curve.

Normally, atrial function can be summarized as follows: reservoir, conduit, and contractile [22,23,24,25,26]. The reservoir function affects the other two by creating a passive gradient between chambers and enhancing atrial contraction through stretching fibers (Frank–Starling law). The atrial work is imaged by two loops in a pressure–volume diagram (Figure 3). During diastasis, when L events appear, the atria act as a passive conduit. Atrial conduit function is affected by atrial compliance, ventricular relaxation, and to a lesser extent by ventricular compliance. On the other hand, factors influencing reservoir function can also indirectly alter the conduit function.

During AF, the atria lack active contraction so they have to work at a higher pressure to fill the ventricles adequately, the conduit phase lengthens, and L events are more likely to take place.

In 1986, Keren et al. studied the relationship between L events and other diastolic events [27]. They stated that rapid early inflow through a low-resistant mitral valve causes a drop in atrial pressure so that ventricular pressure can rapidly increase and produce a reversal gradient stopping the transmitral flow. Reestablishment of a positive atrioventricular pressure gradient secondary to incoming and delayed pulmonary venous flow (PVF) accounts for the L wave and the L motion. They observed in 12 young healthy subjects an almost constant interval between PVF and L events not influenced by RR variations of normal breathing. To corroborate this hypothesis, a low-transient gradient between the left chambers during diastasis was observed invasively in eight conscious dogs. Furthermore, using a computational model of the left cardiac site, they also evaluated the cardiac pressures and gradients in correlation to three different variables, time ventricular relaxation (T), passive elastic constant (α), and transmitral resistance. The used modality was that the variation of each of those variables happened keeping the other two constant. In this model, mid-diastolic transmitral flow was favored by rapid relaxation and increased stiffness, while it was hampered by mitral stenosis or impaired relaxation. The investigators’ model had two main ventricular determinants of L events: a very fast-filling diastolic phase with or without augmented left atrial pressure and increased stiffness. A very rapid early diastolic filling phase is compatible with advanced forms of diastolic dysfunction (grade II and III) or supernormal diastolic patterns (typical of young healthy individuals) as evidenced by the U-shaped relationship between early diastolic peak mitral inflow velocity (E wave) and filling pressure [28]. The main limitation of this model was that ventricular compliance and ventricular relaxation were considered to be two independent diastolic components. Indeed, impaired ventricular compliance is related to altered ventricular relaxation, as evidenced by the progressive reduction of the peak mitral annular early diastolic velocity (E’ wave) (marker of LV relaxation) in worsening diastolic dysfunction from grade I to III.

Contrary to the model of Karen et al., Hatle et al. speculated that delayed left ventricular relaxation could contribute to L wave genesis [29]. Accordingly, most of the subjects with the L wave presented with a low amplitude of the E’ wave and the Valsalva maneuver unmasked an impaired relaxation in many of them [13,20,30]. Moreover, L’ wave temporally preceding (or overlapping) the L wave leads to the hypothesis of an active role of delayed relaxation in the genesis of the L wave. The mechanism at the basis of this phenomenon might be a delayed ventricular diastolic suction effect [20] (described as “post-early diastolic relaxation”).

**Figure 4:**
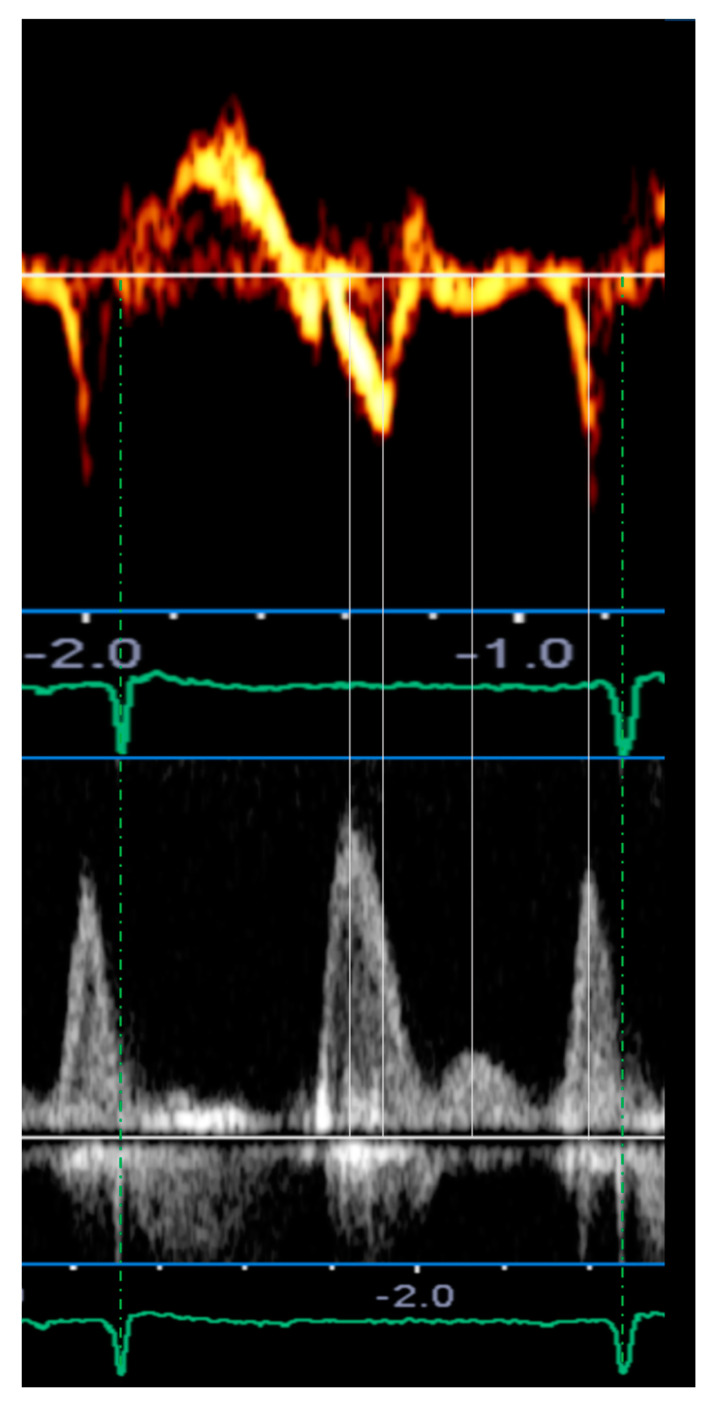
The upper and lower panels show septal mitral annular velocity by tissue Doppler imaging (TDI) and transmitral flow velocity in the pulsed-wave Doppler mode, respectively. The two Doppler spectra relate to two consecutive RR cycles which had the same duration of 1100 ms (dashed green lines), therefore a temporal comparison was possible.

**Figure 5:**
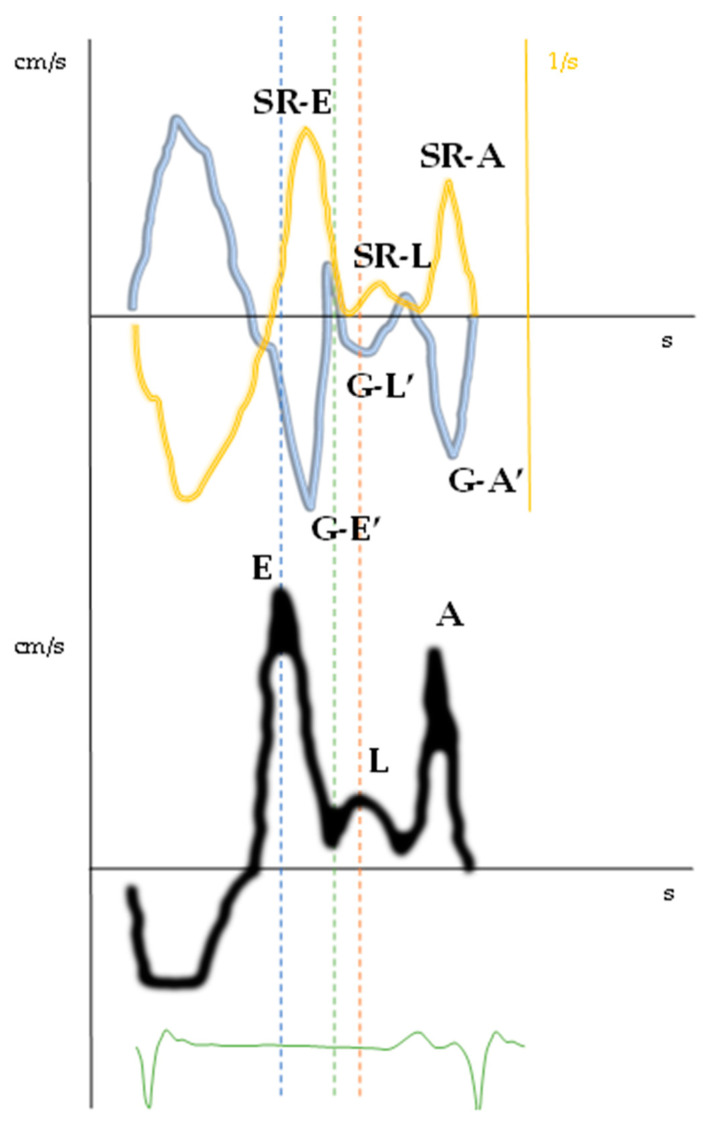
Temporal relationships between velocity, strain rate ventricular curves, and transmitral flow patterns in pulsed-wave Doppler. The upper panel shows the strain rate (yellow) and velocity (blue) ventricular curves. The lower panel shows the spectral Doppler of transmitral flow. The E wave happens before the early diastolic peak velocity and strain rate of the left ventricle. The mid-diastolic events occur simultaneously. SR-E: peak early diastolic strain rate; SR-L: peak mid-diastolic strain rate; SR-A: peak late diastolic strain rate; G–E’: global early diastolic ventricular velocity using STI; G–L’: global mid-diastolic ventricular velocity using STI; G–A’: global late diastolic ventricular velocity using STI; E wave: early diastolic transmitral flow velocity; L wave: mid-diastolic transmitral flow velocity; A wave: late transmitral flow velocity.

In normal individuals, the diastole is initiated by the relaxation of the ventricle through a “ventricular suction mechanism”, thus the E’ wave occurs simultaneously or slightly before the E wave. In case of advanced (diastolic dysfunction) DD, high atrial pressure causes the opening of the mitral leaflets when the ventricle has not yet begun to relax, thus the E wave anticipates the E’ wave (Figure 4). In this regard, we evaluated a patient with a triphasic transmitral pattern to demonstrate the myocardial mechanics of L events through the use of atrial and ventricular speckle tracking imaging (STI) on the same reference heartbeat (Table 3). Specifically, we observed that all the classic echocardiographic parameters linked to advanced diastolic dysfunction were present as dilated LA and increased E/E’ ratio. Moreover, altered early ventricular relaxation and augmented stiffness were confirmed by the low value of the strain rate during isovolumetric relaxation time (SRirt) and the high E wave/SRirt ratio. These parameters correlated with time ventricular relaxation (T) and pulmonary capillary wedge pressure (PCWP) and seemed to be less affected by loading conditions and the effect of tethering or translational motion [31,32,33]. With increased left atrial pressure, the timing of early diastolic events was inverted with the E wave preceding the E’ wave, the global early diastolic ventricular velocity at STI (G-E’), and the peak early diastolic strain rate (SR-E) (Figure 4 and Figure 5, Table 3).

**Figure 6:**
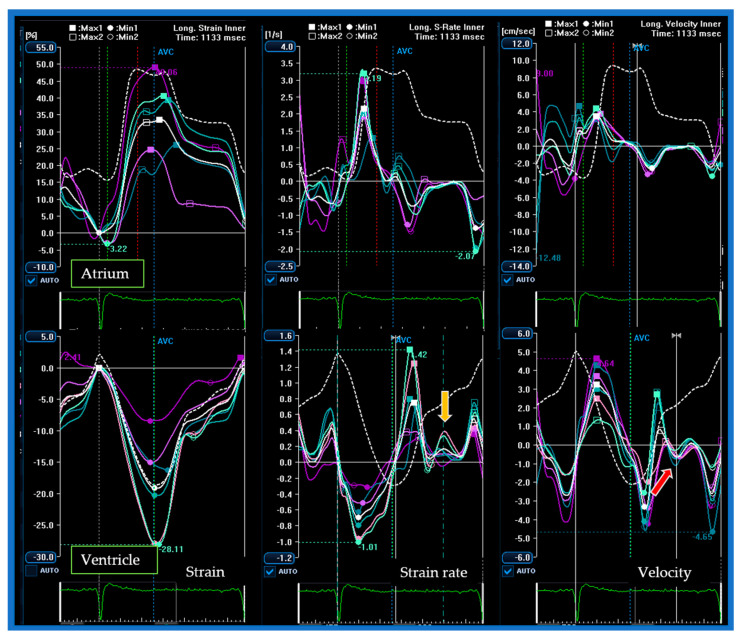
Atrial and ventricular strain, strain rate, and velocity curves obtained from the four-chamber view of the same reference heartbeat. The red arrow indicates the global mid-diastolic ventricular velocity using STI (G–L’). The yellow arrow indicates the peak mid-diastolic strain rate (SR-L). The atrial stain, velocity, and strain rate curves do not show any significant changes in correspondence with mid-diastolic ventricular events.

During diastasis, the LV exhibited global mid-diastolic events appreciable in the velocity and strain rate curves (Figure 6) while the atrial curves did not demonstrate any significant mid-diastolic variation of kinetics or size. After correcting for the R–R interval, ventricular longitudinal velocity (G–L’) and mid-diastolic flow occur synchronously, consistently with a delayed ventricular suction effect due to a post-early diastolic relaxation or a “two-step longitudinal relaxation”.

The literature offers other physiopathological mechanisms of L events as well.

The early diastolic phase was described as composed of two moments, the first due to longitudinal relaxation producing a laminar diastolic flow toward the apex and the second due to radial expansion, creating a convective vortex decelerating the flow. The latter phase is responsible for the descending arm of transmitral early flow velocity (E wave) and may be involved in or affect L events [34]. Ghosh et al. described the L wave as a consequence of the vortex generated by rapid early diastolic flow utilizing echocardiography and phase contrast magnetic resonance imaging (PC-MRI) on 10 young healthy volunteers [35]. Their model predicted the presence of two different morphologies of L waves and the presence of lower-amplitude mid-diastolic transtricuspid flow.

There is a paucity of data on tricuspid L events. We found a report of a young patient with previously treated congenital heart disease who presented with acute clinical signs of peripheral congestion. In addition to high pulmonary pressure, a triphasic transtricuspid pattern and the tricuspid L’ wave were related with the same ventricular alterations connected with the appearance of L events on the left side of the heart: hypertrophic ventricle and diastolic dysfunction [36]. Notably, sampling the transtricuspid inflow pattern is not part of a standard echocardiogram.

In summary, there appears to be a different mechanism generating L events in healthy versus pathologic hearts.

## 4. L Events in Sinus Rhythm

L events characterize abnormal heart properties (particularly LVH) and functions both in SR and AF patients. L wave is a marker of poor prognosis and advanced diastolic dysfunction.

Sinus rhythm allows for easier and more rapid interpretation of mid-diastolic events. Lam et al investigated the prognostic role of L events in two studies using 177 recruited patients with LVH and preserved LVEF [18,20]. L wave appeared to be a valid marker of increased LV filling pressure with a sensitivity and specificity of 94% and 88%, respectively. The authors used the same population to investigate the role of both the L’ wave and the L wave. The L’ wave was sampled at both mitral annular sides (septal and lateral). Based on the presence of L events, they considered three groups of patients: group I (L wave with or without any L’ wave); group II (L’ wave with diphasic transmitral pattern), and group III (without any L events). L’ waves were more prevalent than L waves (30% vs. 20%), mainly the lateral ones, and always sampled in case of a triphasic transmitral pattern. Redefining previous criteria for increased LV filling pressure, they reported a greater sensitivity (74 vs. 66%) but lower specificity (82 vs. 94%) of the L’ wave compared with the L wave in predicting high LA pressure. From group III to group I, a gradient of increasing diastolic dysfunction and increasing incidence of hospitalization for HF during the follow-up were noted. L’ waves seemed to be a valid predictor of future HF and the risk further increased when both L events were observed in the same patient. As expected, the Valsalva maneuver performed on 22 patients led to the disappearance of the L wave but did not modify the L’ wave, which suggests a lesser load dependency of the L’ wave. It also would have been interesting to utilize a provocative maneuver on group II patients to evaluate the possible appearance of a triphasic transmitral pattern and the possible subcategorization of this cohort.

Consistent with the previous study, Ha et al. reported that the L’ wave in addition to the L wave indicated a more advanced diastolic dysfunction [13]. They studied the association between markers of diastolic dysfunction and the presence of septal L’ wave in 83 subjects prospectively enrolled with a triphasic diastolic pattern, 17% of whom had HF. Almost all of them presented some grade of diastolic dysfunction. As above, the septal L’ wave was registered in the majority of patients and related with more severe diastolic dysfunction (e.g., E/E’ ratio >15 was present in 64% of the patients with the L’ wave vs. 36% of the patients without). As reported in AF patients, the N-type natriuretic peptide (NP) levels sampled at the same time of the echocardiogram were significantly higher in the patients with both L events (*p* = 0.0012). The amplitude (*p* = 0.0002) and the time velocity integral (TVI) of the L wave were significantly greater in the patients with the L’ wave (*p* = 0.0079), but the meaning of this remains elusive. The authors utilized the Valsalva maneuver performed in all but two patients and an impaired relaxation pattern was unmasked in 85% of the cases, but no information about L’ wave variations during maneuvers was reported. In partial disagreement with what was previously reported, a prospective study for long-term prognosis involving 144 subjects with a triphasic transmitral pattern reported that the septal L’ wave (noted in about half of the subjects) was more prevalent in the cohort of patients who experienced cardiac events; however, this did not reach statistical significance for predicting clinical outcomes (*p* = 0.059). A particularly poor prognosis was reported among the patients with altered NP, dilated LA, and a triphasic transmitral pattern [12].

The prognostic role of the L wave in HF patients was explored by Masai et al. in 2017 [16]. The studied population was composed of 151 HF patients (69 HFpEF and 82 HFrEF) admitted to the hospital in SR and without any significant VHD. The echocardiography at the time of discharge showed an L wave in 48 subjects. During a median follow-up of 17 months, the L wave-positive group had a poor prognosis independent of LVEF values (Kaplan–Meier analysis *p* < 0.01; *p* < 0.05 for all-cause mortality in HFpEF). In both groups (HFrEF and HFpEF), the presence of the L wave was connected to grade II and III of diastolic dysfunction as well as to major anatomical alteration (as the left ventricle mass or the relative wall thickness (RWT)). The provocative maneuvers (leg lifting or infusion of a normal saline bolus) performed on HFpEF patients evoked the appearance of an L wave suggesting their diastolic equilibrium could be easily unbalanced. Regarding this, in our experience, we observed several cases of L wave and L’ wave appearance after extrasystolic beats (both atrial and ventricular) in decompensated patients without any baseline L events. A post-extrasystolic beat increases venous return and could be a surrogate of the provocative maneuvers providing similar information.

The common final pathway of advanced diastolic dysfunction is the increased LV filling pressure with pulmonary vascular congestion so the aim of diastolic evaluation is to know if the filling pressure is above normal. The connection between L events and increased filling pressure was mostly based on indirect markers, E/E’ ratio, while invasive measurement often failed to demonstrate a significant correlation [10,37,38].

In a publication involving 48 patients affected by dilated cardiomyopathy undergoing catheterization for heart transplantation candidacy, the mean pulmonary capillary wedge pressure (PCWP) was significantly higher in the cohort expressing an L wave (*p* = 0.015). Although with a limited sample size, this was one of the few studies that correlated invasive data with L events. The L’ wave was present in over a quarter of the total population (and in the majority of the patients with the L wave) but without reaching any clinically relevant association. This was also reported in almost half of the healthy volunteers and in this case the authors presumed a different mechanism producing the L’ pattern in healthy versus HF patients [21]. This result is consistent with a previous publication that reported no significant differences in the value of the time constant of the isovolumic LV pressure decline (Ͳ) or PCWP (invasively measured) between the cohort expressing an isolated L’ wave and the one that did not. The presence of a mid-diastolic apical–basal motion (an “inverted L’ wave”) was inversely related with Ͳ and PCWP [38].

## 5. L Events in Atrial Fibrillation

Atrial fibrillation with normal or slow ventricular response represents the best condition to identify L events owing to the absence of atrial systole, the long duration of diastasis, and the frequent coexistence of LVH and diastolic dysfunction. On the other hand, variability of R–R intervals and beat-to-beat changes in loading conditions can lead to difficulties in the interpretation of echocardiographic data. The real-world evaluation of diastolic function in AF patients is often considered time-consuming, not informative, challenging, and in many cases simply labeled as a “monophasic transmitral pattern”. Nonetheless, the L wave could be a simple and informative parameter to consider at the time of diastolic evaluation with incremental prognostic value.

Nakai et al. studied the prognostic impact of the L wave on 99 persistent AF patients during a mean follow-up of 5.7 months [15]. The event rates of hospitalization for HF and cardiac death were significantly higher in the patients presenting with an L wave (18% vs. 6%, *p* = 0.05). The L wave was associated with significant major cardiac morphologic alterations such as LVH (85% vs. 43%, *p* < 0.05), echocardiographic markers of increased left atrial pressure such as E/e’ ratio >15 (71% vs. 31%, *p* < 0.001), as well as higher levels of NP measured together with echocardiograms. No significant correlation was reported between the amplitude of the L wave and the E/e’ ratio or the left atrium volume so the mere identification of the L wave informed about advanced diastolic dysfunction and poor prognosis. Subsequently, consistent results on the prognostic value of the L wave in a similar population of AF patients were reported [17]. The patients were followed for a longer period and the evidence of an L wave was independently associated with increased hospitalization for HF (HR, 2.22; *p* = 0.016) and all-cause mortality (HR, 7.0363; *p* < 0.001). The authors also demonstrated that added to conventional clinical and echocardiographic parameters, the L wave can provide incremental prognostic value for adverse cardiac events (*p* = 0.048) and all-cause mortality (*p* = 0.002).

The natural history of AF contemplates an early stage of paroxysmal episodes that evolve later in the stable stage of persistent AF. This evolution involves structural changes of LA, which appears dilated, fibrotic, and dysfunctional. Recurrence of AF after spontaneous or iatrogenic cardioversion is a frequent problem and can cause episodes of syncope, acute HF, and thromboembolism. Left atrial dimensions, LVH grade, and advanced diastolic dysfunction are well-known risk factors of AF recurrence after electrical cardioversion [39]. Ari et al. investigated the use of the L wave as a marker of arrhythmic recurrence after electrical cardioversion by enrolling 70 subjects with persistent AF [14]. The cohort of patients with the L wave was clinically distinguished by longer-lasting AF (9.0 ± 1.54 months vs. 7.16 ± 2.52 months; *p* < 0.003) and a lower mean ventricular response compared to the other. Consistent with previous studies, the L wave-positive group had significantly greater morphologic cardiac alterations (LA volume and LV mass) and more severe diastolic alteration, as exemplified by the E/e’ ratio or the E/Vp ratio (*p* < 0.001). After achieving SR, the recurrence rate of AF at 30 days was 45% in the L wave-positive group, and the L wave was an independent predictor of early arrhythmia recurrence (OR: 0.29; 95% CI: 0.08–0.98; *p* = 0.04). The control echocardiograms in SR at 24 h after successful ECV showed differences in diastolic parameters but the presence of the L wave was no longer present. It would have been interesting to know how many patients still presented an L wave in SR. This leads us to the question: “Can the evidence of an L wave have prognostic implications even if it only appears in specific circumstances (e.g., atrial fibrillation)?” This assertion seems likely. Another question is if the L wave appearance during provocative maneuvers in an L wave-“naïve” patient would have long-term prognostic implications.

Some interesting single experiences reported the transient appearance of the L wave in different pathologic conditions of volume overload, such as severe anemia, new-onset HF (with or without new-onset AF), acute VHD, or acute coronary syndrome (ACS) [40,41].

Morisawa et al. described an interesting case of a pacemaker (PM)-dependent patient (pacing mode DDD) developing acute HF and new-onset AF [42]. In these circumstances, the device pacing mode automatically switched to VVI. Giant L waves were noted during the echocardiogram. They studied the relationships existing between HR, fluid overload, pacing mode, and amplitude of the L wave. With equal fluid status, the L wave disappeared at different HRs based on the pacing mode (80 beats per minute (bpm) for VVI and 70 bpm for DDD). After restoring SR and improving the congestion state, there was no L wave at 60 bpm in the VVI pacing mode. The authors suggested that the L wave could be a marker of fluid overload; furthermore, its amplitude change may be utilized as a parameter for guiding diuretic treatment. Limitations for this use come from the adaptive tachycardic response in decompensating HF patients due to neurohormonal activation.

Interestingly, another report described by Misumi et al. reported the appearance of an L wave in a patient developing acute severe mitral regurgitation, cardiac decompensation, and a transient episode of AF [43]. After surgical correction of the valve defect, no L wave was found in the absence of significant residual mitral valve stenosis.

A common limitation of all the cited studies was the absence of invasive data. In this regard, Kaitka et al. retrospectively enrolled 48 subjects of the CHATARSIS trial. Twenty-four were in SR, 24—in AF [37]. They underwent echocardiography and invasive catheterization on the same day. The results reported no significant correlation between the presence of an L wave in transmitral diastolic pattern and augmented left ventricle end-diastolic pressures (LVEDP) in both AF and SR cohorts (*p* = 0.28 and *p* = 0.14, respectively). According to this, the L wave could represent a prognostic modifying factor and a complex index related to different variables rather than a diagnostic parameter of diastolic dysfunction.

Based on the available data, patients with AF and diastolic dysfunction presenting an L wave have an increased risk of HF, all-cause mortality, and early recurrence of arrhythmia after a rhythm control strategy. Other investigations in larger cohorts are needed to corroborate these data.

## 6. L Events in Hypertrophic Cardiomyopathy

An abnormal diastolic function is an important determinant of HF in HCM, and AF paroxysms frequently exacerbate symptoms. Noninvasive evaluation of diastolic dysfunction in these patients is complex and less accurate [28]. As mentioned above, the L wave seems to be particularly frequent among this group, but the clinical importance of L events has long been unexplored.

In a recent publication by Saito et al., patients with an L wave had adverse midterm prognosis (mean follow-up of 8.8 years) with significantly higher HCM-related death. The L wave was independently associated with HCM-related death in a multivariable analysis using two different models adjusting for conventional SCD risk factors (HR, 2.90; *p* < 0.001) and for baseline factors (HR, 2.38; *p* = 0.001) [11]. L wave patients were younger, more frequently female, with AF and in a slightly poorer New York Heart Association functional class (NYHA) but the baseline characteristics related to SCD, apical aneurysms, or intraventricular obstruction did not differ significantly between the two cohorts.

The role of the L wave in HCM patients with low HCM risk–SCD score and good functional status [10] was studied. The primary endpoints were SCD and lethal arrhythmias. They reported greater NP levels in the L wave-positive patients but no significant differences neither in echocardiographic (such as the E/e’ ratio or LAVI) nor in invasive diastolic parameters (such as T and dP/dV). During a mean follow-up of 4.7 years, the patients with a triphasic transmitral diastolic pattern had a lower event-free survival rate (logrank: *p* = 0.002), and the L wave was independently related to cardiac events (*p* = 0.03). The receiver operating characteristic (ROC) analysis defined 0.2 m/s as the L wave score cutoff value for cardiac events (AUC: 0.732; sensitivity 57.1%; specificity: 88.4%). Consistently with previous data, the SCD rate in this cohort was 0.46% per year [44].

We found a single publication about the presence of an isolated septal L’ wave in an HCM patient with previous septal myectomy and a restrictive triphasic transmitral pattern [45]. The contrast-enhanced cardiac magnetic resonance showed severe septal scarring corresponding to the region of triphasic mitral annular diastolic motion. The authors suggest that a scarred dysfunctional septum could be evident by the presence of a localized L’ wave. The unilateral presence of a septal L’ wave could be the consequence of improved diastolic function of the lateral wall after the intervention. It would be interesting to see if a scarred septum in an ischemic patient could produce a similar unilateral pattern.

Given the relatively high prevalence in this cohort of subjects, the L wave as other new parameters from other techniques (e.g., CMR with LGE) could be used for identifying a subgroup of patients with increased risk of cardiac events, particularly in the low SCD risk score group (Table 4).

## 7. Conclusions

The L wave is by far the most studied mid-diastolic phenomenon. It can define a category of patients at a particularly increased risk of HF and all-cause mortality, particularly when it is combined with altered NP levels. It could also be considered as a modifying risk factor for SCD in HCM patients. L events should be investigated during routine examinations as it is relatively easy to perform and has prognostic implications in different clinical scenarios.

With the exception of young healthy patients, observation of L events is expected in 1–30% of cases based on the individual’s features. The classic patient for L events is a bradycardic elderly female with multiple CV risk factors with marked LVH and grade II–III diastolic dysfunction.

The genesis of L events should be found in the complex atrioventricular interactions. The evaluation of the longitudinal component of diastolic ventricular relaxation with TDI leads to mid-diastolic flow as a consequence of marked delayed ventricular relaxation. In our report, we examined the temporal relationship between transmitral flow and chamber diastolic events. We noted an inverted time of early diastolic events, compatible with a high atrial pressure, followed by synchronous mid-diastolic events, supporting the idea of a “two-step ventricular relaxation” for the genesis of the L wave. The LA curves obtained by STI showed no significant variations during diastasis suggesting a minor role for the LA.

The association of L events with certain anatomical features or changes in loading conditions is consistent with more than one theory and the acceptance of one does not strictly exclude the other so it appears there is a different mechanism generating L events in healthy versus pathological hearts.

In the acute context, such as acute VHD or ACS, sudden and reversible appearance of L events is mainly related to an abrupt elevation of filling pressure. Therefore, in decompensated patients, the evidence of an L wave could contribute to the assessment of loading conditions and response to diuretic treatment.

Mid-diastolic mitral annular motion is frequent in healthy subjects without clear meaning, especially if not accompanied by a triphasic transmitral pattern, but could represent a diastolic alteration preceding the appearance of an L wave.

Further studies including L events in prognostic risk scores in specific subsets of patients are warranted to assess their clinical significance.

## Figures and Tables

**Table 1:** Mid-diastolic events (L events).

Type	Definition	Sampling Site and Method
Mid-diastolic transmitral flow velocity (L wave)	Distinct forward flow velocity after E wave with peak velocity ≥ 20 cm/s	Apical four-chamber view, sample volume at the tips of mitral leaflets with PW Doppler
Mid-diastolic mitral valve motion (L motion)	Mid-diastolic opening and closing motion of the mitral valve	Parasternal long-axis view M-mode along the line-cutting mitral valve
Mid-diastolic mitral annular velocity (L’ wave)	Distinct basilar–apical tissue velocity after E’ wave present in all cardiac cycles *	Apical four-chamber view, sample volume at septal and lateral mitral annular corners (TDI and PW Doppler)

E’ wave (or e’ wave): early diastolic transmitral flow velocity. PW: pulsed-wave Doppler mode. M-mode: monodimensional mode. TDI: tissue Doppler imaging. * In some publications it was defined as basilar–apical mid-diastolic annular velocity > 20 cm/s.

**Table 3:** Echocardiography parameters.

Doppler-Parameters	Values	Chambers	Values	Strain and Strain Rate Parameters of LV and LA	Values	Time to Peak (ms)	%RR
E wave (cm/s)	119.8	IVS (mm)	11	GLS (%)	−16.7	E wave	49
L wave (cm/s)	32.3	EDD (mm)	53	SR-irt (1/s)	0.06	G–E’	53
A wave (cm/s)	100.6	EDV (mL/m^2^)	63.9	SR-E (1/s)	0.69	SR-E	56
E/A ratio	1.2	ESV (mL/m^2^)	23.3	E/SR-ivrt ratio (cm)	1997	G–L’	73
E’ lat wave (cm/s)	8.2	LVEF (%)	63	E/SR-E ratio (cm)	1.7	L wave	73
L’ lat wave (cm/s)	3.0	LAVi (mL/m^2^)	42.2	SR-L (1/s)	0.13	SR-L	75
A’ lat wave (cm/s)	13.8			G–L’ (cm/s)	0.59		
E’ ivs wave (cm/s)	8.1			LA strain reservoir (%)	34.2		
L’ ivs wave (cm/s)	2.2			LA strain conduit (%)	13.6		
A’ ivs wave (cm/s)	5.8			LA strain contraction (%)	20.5		
E/E’	14.7			aSR-E (1/s)	−0.77		
IVRT (PW) (ms)	61			aSR-A (1/s)	−1.39		
DT (ms)	178			(E/E’)/LA strain reservoir	0.44		
S/D wave ratio	<1						
TRV (m/s)	2.8						

This table refers to a seventy-seven-year old man with multiple cardiovascular risk factors, polyvasculopathies, high level of NT-proBNP values, and signs of postcapillary hypertension with a normal cardiothoracic ratio. At chest X-ray, normal-sized, normokinetic LV, mildly dilated left atrium, absence of significant valvulopathies with advanced diastolic dysfunction and triphasic transmitral pattern at echocardiographic color Doppler evaluation underwent coronary angiography followed by angioplasty with stenting of the circumflex artery. The STI study of the LV showed an initial global systolic dysfunction with a mildly reduced GLS and global longitudinal diastolic dysfunction. The isovolumetric relaxation time was calculated using PW Doppler. The intermediate time between aortic valve closure and the end of IVRT was used for calculating the global longitudinal strain rate during SR-irt. SR-E and SR-irt were reduced and the derived ratios E/SR-E and E/SR-ivt were altered. Even the atrial function was mildly abnormal, with reduced LA strain reservoir and conduit, while the contractile function was preserved. The time to peak values are normalized for the R–R intervals. E wave: early diastolic transmitral flow velocity; L wave: mid-diastolic transmitral flow velocity; A wave: late transmitral flow velocity; e’ wave: early diastolic mitral annular velocity; L’ wave: mid-diastolic mitral annular velocity; a’ wave: late diastolic mitral annular velocity; IVRT: isovolumetric relaxation time; DT: deceleration time; TVR: tricuspid regurgitant jet velocity; IVS: interventricular septum; EDD: end-diastolic diameter; EDV: end-diastolic volume; ESV: end-systolic volume; LVEF: left ventricle ejection fraction; LAVi: left atrial volume index; GLS: global longitudinal strain; SRirt: strain rate at the isovolumetric relaxation time; SR-E: peak early diastolic strain rate; SR-L: peak mid-diastolic strain rate; G–L’: global mid-diastolic ventricular velocity using STI. G–E’: global early diastolic ventricular velocity using STI; aSR-E: atrial peak early diastolic strain rate; aSR-A: atrial peak late diastolic strain rate; E/e’/LA strain reservoir: left atrial stiffness.

**Table 4:** Diagnostic and prognostic value of mid-diastolic events in the main clinical settings.

Relevant Clinical Associations	Clinical Settings			
	Aymptomatic DD	HFrEF/HFpEF	HCM	NVAF
**Diagnostic Value**				
BNP/NT-proBNP serum levels elevation	+			+
Grade II-III diastolic dysfunction	+	+	+	+
**Prognostic Value**				
Hospitalization for HF or new-onset HF	+	+	+	+
Cardiovascular mortality	+	+		+
All-cause mortality	+	+		
SCD, VAs			+	
AF or Recurrent AF after ECV			+	+

AF: atrial fibrillation; BNP: brain natriuretic peptide; DD: diastolic dysfunction; ECV: electrical cardioversion; HF: heart failure; HFpEF: heart failure with preserved ejection fraction; HFrEF: heart failure with reduced ejection fraction; NVAF: nonvalvular atrial fibrillation; NT-proBNP: N-terminal pro b-type natriuretic peptide; SCD: sudden cardiac death; VAs: ventricular arrhythmias.

## References

[1] Muscogiuri G., Chiesa M., Trotta M., Gatti M., Palmisano V., Dell’Aversana S., Baessato F., Cavaliere A., Cicala G., Loffreno A. (2020). Performance of a deep learning algorithm for the evaluation of CAD-RADS classification with CCTA. Atherosclerosis.

[2] Pontone G., Guaricci A.I., Palmer S.C., Andreini D., Verdecchia M., Fusini L., Lorenzoni V., Guglielmo M., Muscogiuri G., Baggiano A. (2020). Diagnostic performance of non-invasive imaging for stable cor-onary artery disease: A meta-analysis. Int. J. Cardiol..

[3] Guglielmo M., Fusini L., Muscogiuri G., Baessato F., Loffreno A., Cavaliere A., Rizzon G., Baggiano A., Rabbat M.G., Muratori M. (2021). T1 mapping and cardiac magnetic resonance feature tracking in mitral valve prolapse. Eur. Radiol..

[4] Guaricci A.I., Masci P.G., Muscogiuri G., Guglielmo M., Baggiano A., Fusini L., Lorenzoni V., Martini C., Andreini D., Pavon A.G. (2021). CarDiac magnEtic Resonance for prophylactic Implantable-cardioVerter defibrillAtor ThErapy in Non-Ischaemic dilated CardioMyopathy: An international Registry. Europace.

[5] Muscogiuri G., Martini C., Gatti M., Dell’Aversana S., Ricci F., Guglielmo M., Baggiano A., Fusini L., Bracciani A., Scafuri S. (2021). Feasibility of late gadolinium enhancement (LGE) in ischemic cardiomyopathy using 2D-multisegment LGE combined with artificial intelligence reconstruction deep learning noise reduction algorithm. Int. J. Cardiol..

[6] Muscogiuri G., Fusini L., Ricci F., Sicuso R., Guglielmo M., Baggiano A., Gasperetti A., Casella M., Mushtaq S., Conte E. (2021). Additional diagnostic value of cardiac magnetic resonance feature tracking in patients with biopsy-proven arrhythmogenic cardiomyopathy. Int. J. Cardiol..

[7] Gaibazzi N., Porter T., Lorenzoni V., Pontone G., de Santis D., de Rosa A., Guaricci A.I. (2017). Effect of Coronary Revascular-ization on the Prognostic Value of Stress Myocardial Contrast Wall Motion and Perfusion Imaging. J. Am. Heart. Assoc..

[8] Badano L.P., Caravita S., Rella V., Guida V., Parati G., Muraru D. (2021). The Added Value of 3-Dimensional Echocardiography to Understand the Pathophysiology of Functional Tricuspid Regurgitation. JACC Cardiovasc. Imaging.

[9] Edler I. (2009). Part Three:Atrioventricular Valve Motility In The Living Human Heart Recorded By Ultrasound. Acta Medica Scand..

[10] Sugiura Y., Morimoto R., Aoki S., Yamaguchi S., Haga T., Kuwayama T., Yokoi T., Hiraiwa H., Kondo T., Watanabe N. (2019). Prognostic impact of mitral L-wave in patients with hypertrophic cardiomyopathy without risk factors for sudden cardiac death. Heart Vessel..

[11] Saito C., Minami Y., Arai K., Haruki S., Shirotani S., Higuchi S., Ashihara K., Hagiwara N. (2020). Prognostic Significance of the Mitral L-Wave in Patients with Hypertrophic Cardiomyopathy. Am. J. Cardiol..

[12] Kim S.-A., Son J., Shim C.-Y., Choi E.-Y., Ha J.-W. (2017). Long-term outcome of patients with triphasic mitral flow with a mid-diastolic L wave: Prognostic role of left atrial volume and N-terminal pro-brain natriuretic peptide. Int. J. Cardiovasc. Imaging.

[13] Ha J.W., Ahn J.A., Moon J.Y., Suh H.S., Kang S.M., Rim S.J., Jang Y., Chung N., Shim W.H., Cho S.Y. (2006). Triphasic mitral inflow velocity with mid-diastolic flow: The presence of mid-diastolic mitral annular velocity indicates advanced diastolic dys-function. Eur. J. Echocardiogr..

[14] Ari H., Ari S., Akkaya M., Sarigul O.Y., Emlek N., Aydin C., Çetinkaya S., Bozat T., Melek M. (2013). A novel echocardio-graphic predictor of atrial fibrillation recurrence: L-wave. Echocardiography.

[15] Nakai H., Takeuchi M., Nishikage T., Nagakura T., Otani S. (2007). The Mitral L Wave A Marker of Advanced Diastolic Dysfunction in Patients with Atrial Fibrillation. Circ. J..

[16] Masai K., Mano T., Goda A., Sugahara M., Daimon A., Asakura M., Ishihara M., Masuyama T. (2018). Correlates and Prognostic Values of Appearance of L Wave in Heart Failure Patients with Preserved vs. Reduced Ejection Fraction. Circ. J..

[17] Su H.-M., Lin T.-H., Hsu P.-C., Lee W.-H., Chu C.-Y., Lee C.-S., Lai W.-T., Sheu S.-H., Voon W.-C. (2013). Incremental prognostic value of identifying mitral L wave in patients with atrial fibrillation. Int. J. Cardiol..

[18] Lam C.S., Han L., Ha J.-W., Oh J.K., Ling L.H. (2005). The mitral L wave: A marker of pseudonormal filling and predictor of heart failure in patients with left ventricular hypertrophy. J. Am. Soc. Echocardiogr..

[19] Ha J.-W., Oh J.K., Redfield M.M., Ujino K., Seward J.B., Tajik A. (2004). Triphasic mitral inflow velocity with middiastolic filling: Clinical implications and associated echocardiographic findings. J. Am. Soc. Echocardiogr..

[20] Lam C.S., Han L., Oh J.K., Yang H., Ling L.H. (2008). The Mitral Annular Middiastolic Velocity Curve: Functional Correlates and Clinical Significance in Patients with Left Ventricular Hypertrophy. J. Am. Soc. Echocardiogr..

[21] Podroužková M.J., Špinarová H., Hude P., Krejčí J. (2013). flow and mid- diastolic mitral annular motion—Relation to pulmonary capillary wedge pressure in dilated cardiomyopathy patients. Kardiol. Rev..

[22] Guglielmo M., Baggiano A., Muscogiuri G., Fusini L., Andreini D., Mushtaq S., Conte E., Annoni A.D., Formenti A., Mancini E.M. (2019). Multimodality imaging of left atrium in patients with atrial fibrillation. J. Cardiovasc. Comput. Tomogr..

[23] Cameli M., Sciaccaluga C., Loiacono F., Simova I., Miglioranza M.H., Nistor D.O., Bandera F., Emdin M., Giannoni A., Ciccone M.M. (2019). The analysis of left atrial function predicts the severity of functional impairment in chronic heart failure: The FLASH multicenter study. Int. J. Cardiol..

[24] Pontone G., Andreini D., Bertella E., Petullà M., Russo E., Innocenti E., Mushtaq S., Gripari P., Loguercio M., Segurini C. (2015). Comparison of cardiac computed tomography versus cardiac magnetic resonance for characterization of left atrium anatomy before radiofrequency catheter ablation of atrial fibrillation. Int. J. Cardiol..

[25] Barbier P., Solomon S.B., Schiller N.B., Glantz S.A. (1999). Left Atrial Relaxation and Left Ventricular Systolic Function Determine Left Atrial Reservoir Function. Circulation.

[26] Hoit B.D., Shao Y., Gabel M., Walsh R.A. (1994). In vivo assessment of left atrial contractile performance in normal and patho-logical conditions using a time-varying elastance model. Circulation.

[27] Keren G., Meisner J.S., Sherez J., Yellin E.L., Laniado S. (1986). Interrelationship of mid-diastolic mitral valve motion, pulmonary venous flow, and transmitral flow. Circulation.

[28] Nagueh S.F., Smiseth O.A., Appleton C.P., Byrd B.F., Dokainish H., Edvardsen T., Flachskampf F.A., Gillebert T.C., Klein A.L., Lancellotti P. (2016). Recommendations for the Evaluation of Left Ventricular Diastolic Function by Echocardiography: An Update from the American Society of Echocardiography and the European Association of Cardiovascular Imaging. J. Am. Soc. Echocardiogr..

[29] Hatle L. (1993). Doppler echocardiographic evaluation of diastolic function in hypertensive cardiomyopathies. Eur. Heart J..

[30] Masai M.T., Mano T., Goda A., Soyama Y., Matsumoto A., Masuyama T. (2016). Clinical Significance of Mid-Diastolic L Wave in Heart Failure Patients by Various Loading Test. J. Card. Fail..

[31] Wang J., Khoury D.S., Thohan V., Torre-Amione G., Nagueh S.F. (2007). Global Diastolic Strain Rate for the Assessment of Left Ventricular Relaxation and Filling Pressures. Circulation.

[32] Choudhury A., Magoon R., Malik V., Kapoor P.M., Ramakrishnan S. (2017). Studying diastology with speckle tracking echo-cardiography: The essentials. Ann. Card. Anaesth..

[33] Kasner M., Gaub R., Sinning D., Westermann D., Steendijk P., Hoffmann W., Schultheiss H.-P., Tschöpe C. (2010). Global strain rate imaging for the estimation of diastolic function in HFNEF compared with pressure-volume loop analysis. Eur. J. Echocardiogr..

[34] Tanaka M., Sakamoto T., Sugawara S., Nakajima H., Kameyama T., Tabuchi H., Katahira Y., Ohtsuki S., Kanai H. (2011). Physi-ological basis and clinical significance of left ventricular suction studied using echo-dynamography. J. Cardiol..

[35] Ghosh E., Caruthers S.D., Kovács S.J. (2014). E-wave generated intraventricular diastolic vortex to L-wave relation: Model-based prediction with in vivo validation. J. Appl. Physiol..

[36] Hayabuchi Y., Ono A., Homma Y., Kagami S. (2016). Tricuspid L and L’ waves. Int. J. Cardiol..

[37] Koitka K., Kelly N., Lau K., Lin A., Chan J., Scalia G., Hamilton-Craig C. (2018). Does Mid-Diastolic Transmitral Flow (‘L-wave’) Correlate with Raised Left Ventricular End Diastolic Pressure. Heart Lung Circ..

[38] Su H.-M., Lin T.-H., Lee C.-S., Lee P.-C., Yeh S.-C., Chen W.-R., Lai W.-T., Sheu S.-H., Voon W.-C. (2008). Mid-Diastolic Mitral Annular Motion: A Useful Marker in the Evaluation of Left Ventricular Relaxation and End-Diastolic Pressure. Ultrasound Med. Biol..

[39] Ecker V., Knoery C., Rushworth G., Rudd I., Ortner A., Begley D., Leslie S.J. (2018). A review of factors associated with maintenance of sinus rhythm after elective electrical cardioversion for atrial fibrillation. Clin. Cardiol..

[40] Misumi I., Ishii M. (2017). Triphasic mitral inflow pattern associated with hemodynamic deterioration in anemia or mitral regurgitation: A report of two cases. J. Med. Ultrason..

[41] Misumi I., Ebihara K., Akahoshi R., Rokutanda T., Uramoto H., Esaki T., Matsumoto M., Sugiyama S., Ogawa H. (2011). Triphasic mitral inflow in acute coronary syndrome: A case study. J. Cardiol. Cases.

[42] Morisawa D., Ohno Y., Ohta Y., Orihara Y., Masai K., Goda A., Asakura M., Ishihara M. (2019). Serial changes of L wave according to heart rates in a heart failure patient with persistent atrial fibrillation. J. Cardiol. Cases.

[43] Misumi I., Motozato K., Usuku H., Sakamoto K., Kaikita K., Tsujita K., Fukui T. (2019). A Mechanism for L-Wave Generation via Color M-Mode Imaging in a Patient with Mitral Regurgitation. CASE.

[44] Maron B.J., Rowin E.J., Casey S.A., Link M.S., Lesser J.R., Chan R.H., Garberich R.F., Udelson J.E., Maron M.S. (2015). Hypertrophic Cardiomyopathy in Adulthood Associated With Low Cardiovascular Mortality with Contemporary Management Strategies. J. Am. Coll. Cardiol..

[45] Debl K., Djavidani B., Buchner S., Poschenrieder F., Feuerbach S., Riegger G., Luchner A. (2008). Triphasic Mitral Inflow Pattern and Regional Triphasic Mitral Annulus Velocity in Hypertrophic Cardiomyopathy. J. Am. Soc. Echocardiogr..

